# The mediating role of alexithymia in the relationship between childhood trauma and exercise addiction

**DOI:** 10.3389/fpsyg.2026.1803371

**Published:** 2026-05-18

**Authors:** Hasan Ünver, Pınar Demir Gündoğmuş, Evrim Bayrak Oruç, Safiye Zeynep Tatlı, Mehmet Rıdvan Varlı, Mehmet Ali Özdemir, Baran Kocaman, İbrahim Gündoğmuş, Yasir Şafak

**Affiliations:** 1 Department of Psychiatry, Ankara Etlik City Hospital, Ankara, Türkiye; 2 Department of Sports Medicine, Gulhane Training and Research Hospital, Ankara, Türkiye; 3 Mood Disorders Program, UH Cleveland Medical Center, Cleveland, OH, United States; 4 Department of Sports Medicine, Ankara University Faculty of Medicine, Ankara, Türkiye

**Keywords:** alexithymia, childhood trauma, emotion processing, exercise addiction, exercise dependence

## Abstract

**Background:**

Childhood trauma (CT) is a risk factor for addictive behaviors, including exercise addiction (EA). Alexithymia—difficulties in identifying and describing emotions—is associated with both CT and addiction. This study tested whether alexithymia mediates the relationship between CT and EA.

**Methods:**

In this cross-sectional study, 301 adults who exercised regularly (≥6 h/week) completed the Exercise Dependence Scale-21 (EDS-21), Toronto Alexithymia Scale-20 (TAS-20), and Adverse Childhood Experiences Questionnaire (ACE-Q). Group comparisons (alexithymia vs. non-alexithymia), Pearson correlations, and regression-based mediation analyses were performed.

**Results:**

Participants with alexithymia (*n* = 102) had significantly higher EDS-21 total and subscale scores than those without alexithymia (*n* = 199) (all *p* < 0.001). ACE-Q total scores did not differ significantly between groups, but were positively correlated with TAS-20 total score and difficulty identifying feelings (DIF). EDS-21 total and subscale scores were positively correlated with DIF, whereas correlations with externally oriented thinking (EOT) were generally weak and non-significant. In the mediation model, ACE-Q total score was significantly associated with EDS-21 total score (*β* = 0.180, *p* = 0.002), but this association no longer remained significant after TAS-20 total score was entered as a mediator (*β* = 0.090, *p* = 0.133). The indirect effect of ACE-Q on EDS-21 through TAS-20 was significant based on bias-corrected bootstrapped 95% confidence intervals.

**Conclusion:**

Alexithymia statistically mediated the association between childhood trauma and exercise addiction severity in regularly exercising adults. These findings suggest that emotion-processing deficits may represent one pathway linking early adversity to maladaptive exercise patterns. Assessment of exercise addiction may therefore benefit from considering both alexithymic traits and childhood trauma history.

## 1 Introduction

Regular exercise encompasses planned, repetitive physical activity performed with defined frequency, intensity, and duration, and is widely recognized as a key contributor to a healthy lifestyle (Waddington and Smith, 2013). It reduces the risk of lifestyle-related diseases, improves physical capabilities, and enhances quality of life and psychological well-being (Ekelund et al., 2016). By the 1970s, “positive addiction” was introduced to describe increasing amounts and durations of exercise (Glasser, 1976). However, studies have shown that excessive exercise may lead to physical injuries and significant problems in work, family life, and interpersonal relationships (Morgan, 1979). Over the past decade, the concept of exercise addiction (EA) has gained attention as a growing problem that causes serious functional impairments (Weinstein and Szabo, 2023). EA is a dysfunctional behavior characterized by excessive exercise, lack of control over exercise habits, and negative physical, psychological, and social consequences (Szabo and Demetrovics, 2022). Similar to other forms of addiction, EA has six components: salience, tolerance, mood modification, withdrawal, conflict, and relapse. A meta-analysis of 13 studies involving 3,635 participants found that the overall prevalence rate of EA is 6.2% (Weinstein and Szabo, 2023).

Alexithymia is a psychiatric construct characterized by difficulty identifying and processing emotions, difficulty distinguishing emotions from bodily sensations, limited imagination, and a concrete, externally oriented thinking style (Luminet and Nielson, 2025). In the last decade, research on alexithymia has increasingly focused on its relationship with behavioral addiction. It has been proposed that individuals with high alexithymia may engage in addictive behaviors to regulate their emotions (Orsolini, 2020). In contrast, those with lower levels of alexithymia, who experience less difficulty in emotion regulation, may be more successful in managing addictive behaviors (Ghorbani et al., 2017). Associations between alexithymia and pathological gambling (Bonnaire et al., 2017), internet addiction (De Berardis et al., 2009), and other addictive behaviors have been reported. In the exercise domain, swimmers who trained for 22 h per week exhibited higher alexithymia scores than those who trained for only 6 h per week (Allegre et al., 2007).

One plausible mechanism linking alexithymia to EA is impaired emotion regulation. Individuals with alexithymic traits may have difficulty identifying and differentiating internal affective states and may therefore rely more heavily on external regulatory strategies. Within this framework, exercise may serve as a readily available means of mood modification, tension reduction, or escape from poorly differentiated distress. When repeatedly used for this purpose, an initially adaptive behavior may become rigid, excessive, and addiction-like. Emerging evidence suggests that this association may also be shaped by related processes such as interoceptive difficulties, reward sensitivity, and body-related concerns, pointing to a multifaceted pathway through which alexithymic traits may contribute to maladaptive exercise patterns (Bernstein and McNally, 2018; Gori et al., 2021; Lyvers et al., 2021; Sweetnam and Flack, 2023; González-Roz et al., 2024).

Trauma is characterized as a perceived experience that threatens physical integrity and provokes feelings of fear, terror, and helplessness (Carlson and Dalenberg, 2000). Exposure to trauma is common and affects individuals regardless of gender, age, race, ethnicity, or sexual orientation (Reisner et al., 2016). Traumatic experiences may include abuse, violence, neglect, loss, accidents, natural disasters, war, and other emotionally damaging situations (Chrisman and Dougherty, 2014). Studies indicate that adults who experienced trauma during childhood are more susceptible to physical and psychological problems (Noteboom et al., 2021). Childhood trauma (CT) has been associated with a range of psychiatric conditions, including attention deficit/hyperactivity disorder, depression and anxiety disorders, and personality pathology (Peleikis et al., 2022). One study reported higher severity of CT and lifetime trauma exposure in individuals with opioid use disorder (Garami et al., 2019). There is growing interest in exploring the relationship between behavioral addictions and CT. Addictions such as internet/gaming addiction, compulsive sexual behavior, and compulsive buying have been associated with CT (Meng et al., 2024; Slavin et al., 2020; Sansone et al., 2013). As this is a relatively new area of research, there are limited studies examining the relationship between EA and CT. A recent study found higher CT scores among adults at risk for EA (Colledge et al., 2022). CT has been shown to be closely associated with impairments in basic emotional and cognitive processes (Paulus et al., 2021), leading to growing interest in its relationship with alexithymia. The relationship between alexithymia and the emotional neglect and abuse subtypes is particularly prominent (Kick et al., 2024). Studies in adolescents have associated CT with both internet addiction and e-cigarette use, and alexithymia has been proposed as one process that may help explain these links (Wang et al., 2025; Sutherland et al., 2022).

Despite growing interest in EA, important conceptual and methodological gaps remain. Much of the literature has focused on prevalence, descriptive correlates, or bivariate associations rather than explanatory pathways (Weinstein and Szabo, 2023). Current evidence indicates that alexithymia is positively associated with EA (Gori et al., 2021; Morie et al., 2016; Lyvers et al., 2021; Sweetnam and Flack, 2023; González-Roz et al., 2024), while childhood maltreatment is consistently associated with alexithymia and broader psychopathology (Kick et al., 2024; Ditzer et al., 2023; Cerqueira and Almeida, 2023; Yang et al., 2024; Cooper et al., 2024; Karaca Dinç et al., 2021). However, these constructs have rarely been examined in a single model among regularly exercising adults Arayici and Tekinsav Sutcu, 2025). Accordingly, the present study examined whether alexithymia statistically mediates the association between childhood trauma and exercise addiction severity in adults who exercise regularly.

## 2 Methods

### 2.1 Participants

Data were collected between December 2025 and January 2026 in Ankara, Türkiye, using a cross-sectional observational design. The sample comprised volunteer adults aged 18–65 who attended public or private sports centers and reported exercising ≥6 h per week for at least the past 6 months. Written informed consent was obtained from all participants.

Exclusion criteria were self-reported past psychiatric disorder, current psychotropic medication use, any medical or neurological condition that could impair the capacity to provide informed consent or affect reality testing, and incomplete questionnaires. No structured psychiatric interview was conducted. Instead, psychiatric eligibility was assessed during the collection of sociodemographic data, and participants reporting a past psychiatric disorder or current psychotropic medication use were excluded from the study. Ethical approval was obtained from the Ankara Etlik City Hospital Institutional Ethics Committee (AEŞH-BADEK1—2025-674; 17.12.2025), and the study procedures complied with the Declaration of Helsinki.

### 2.2 Measures

Participants completed a questionnaire battery comprising a sociodemographic form, the Exercise Dependence Scale-21 (EDS-21), the Toronto Alexithymia Scale-20 (TAS-20), and the Adverse Childhood Experiences Questionnaire (ACE-Q). Clinical assessments based on the scales were reviewed by two experienced psychiatrists.

#### 2.2.1 Sociodemographic and exercise history

A structured sociodemographic data form developed by the researchers was used to collect information on participants’ demographic and clinical profiles, as well as detailed exercise information. This included the type of sport, duration of participation, weekly frequency, and amateur/professional status.

#### 2.2.2 Exercise dependence scale-21 (EDS-21)

The EDS-21 is a 21-item self-report measure used to evaluate symptoms of EA. Items are rated on a 6-point Likert scale ranging from 1 (“never”) to 6 (“always”). The scale yields a total score as well as subscale scores reflecting core features of behavioral addiction, including withdrawal, persistence, tolerance, loss of control, reduction in other activities, time devoted to exercise, and intention effects (Hausenblas and Downs, 2002). Individuals who score 5 or 6 on the criterion items are classified as exercise dependent, whereas those scoring between 3 and 4 are considered symptomatic and regarded as being at risk for EA. Those scoring 1 or 2 are considered asymptomatic. The scale also includes items that evaluate the physiological and behavioral components of exercise dependence. A validity and reliability study of the Turkish adaptation of the EDS-21 was conducted. In this form, subscores are time and exercise preference, lack of control, withdrawal effects, tolerance, and continuance (Gürbüz and Aşçı, 2006).

#### 2.2.3 Toronto alexithymia scale–20 (TAS-20)

Alexithymia was assessed using the TAS-20, a widely used 20-item self-report instrument (Bagby et al., 1994a, 1994b). Items are rated on a 5-point Likert scale ranging from 1 (strongly disagree) to 5 (strongly agree). The TAS-20 assesses a total score and three alexithymia dimensions: difficulty identifying feelings (DIF), difficulty describing feelings (DDF), and externally oriented thinking (EOT). A TAS-20 score≥61 was used as the criterion for identifying individuals with alexithymia. The Turkish validity and reliability study of the TAS-20 was conducted in 2009 (Güleç et al., 2009).

#### 2.2.4 Adverse childhood experiences questionnaire (ACE-Q)

CT exposure was assessed using the 10-item ACE-Q (Felitti et al., 1998). Participants indicated *yes* or *no* to whether they had experienced specific categories of abuse, neglect, and household dysfunction before the age of 18. Total scores range from 0 to 10, and no cutoff value has been established for the scale. The Turkish version was first translated by Ulukal et al. in 2013, and its validity and reliability were established in 2018 (Gündüz et al., 2018).

### 2.3 Statistical analysis

Statistical analyses were performed using IBM SPSS Statistics version 23.0, and mediation analysis was conducted using AMOS. Before the main analyses, assumptions for parametric and regression-based procedures were evaluated using both statistical tests and visual diagnostics (Ghasemi and Zahediasl, 2012; Ernst and Albers, 2017). Normality was assessed using the Shapiro–Wilk test together with inspection of histograms and Q–Q plots. Linearity and homoscedasticity were evaluated using scatterplots of residuals against predicted values. Multicollinearity was examined using tolerance and variance inflation factor (VIF) values. For between-group comparisons, homogeneity of variance was tested with Levene’s test. No serious violations were identified; therefore, the planned parametric analyses were retained.

Descriptive statistics are presented as mean ± standard deviation (SD) for normally distributed variables and median (interquartile range, IQR) for non-normally distributed variables. Categorical variables are summarized as frequencies and percentages. Participants were categorized into alexithymia (TAS-20 total ≥ 61) and non-alexithymia (TAS-20 total < 61). Between-group differences in sociodemographic and clinical variables were examined with independent-samples t tests or Mann–Whitney U tests for continuous variables, and with chi-square or Fisher’s exact tests for categorical variables, as appropriate. Associations between EA severity (EDS-21), alexithymia dimensions (TAS-20 subscales and total), and ACE (ACE-Q total) were examined using Pearson correlation analyses.

To test the primary hypothesis, a mediation analysis was conducted with adverse childhood experiences (ACE-Q total score) as the independent variable, exercise addiction severity (EDS-21 total score) as the dependent variable, and alexithymia (TAS-20 total score) as the mediator. Unstandardized (B) and standardized (*β*) coefficients were estimated for the total effect (c), direct effect (c′), and indirect effect (a × b). The significance of the indirect effect was tested using a bootstrapping procedure with 5,000 resamples (Preacher and Hayes, 2008). Indirect effects were considered statistically significant if the 95% bootstrap confidence intervals did not include zero. Statistical significance was set at *p* < 0.05 for all analyses.

A post-hoc power analysis was conducted using G*Power (version 3.1) for linear multiple regression with two predictors, based on a medium effect size (*f^2^* = 0.15) and *α* = 0.05. With the achieved sample size of *n* = 301, the estimated statistical power exceeded 0.99, indicating that the sample was adequate to detect medium-sized effects.

## 3 Results

### 3.1 Demographic and exercise-related characteristics

The sociodemographic and exercise-related characteristics of the participants, stratified by alexithymia status, are presented in Table 1. The study sample consisted of 301 total participants, of whom 102 (33.9%) met the criteria for alexithymia, and 199 (66.1%) did not.

**Table 1:** Comparison of socio-demographic characteristics of the participants according to alexithymia.

Variable	Total participant (*n* = 301)	Alexithymia		*χ^2^*/t value	df value	*p* value
		No (*n* = 199)	Yes (*n* = 102)			
Age, years (mean ± SD)	29.0 ± 7.9	29.6 ± 8.3	27.9 ± 6.8	1.714	299	0.088
Education level, *n* (%)				1.376	3	0.711
Primary school	4 (1.3)	2 (50.0)	2 (50.0)			
High school	47 (15.6)	34 (72.3)	13 (27.7)			
University	163 (54.2)	106 (65.0)	57 (35.0)			
Postgraduate	87 (28.9)	57 (65.5)	30 (34.5)			
Sex, *n* (%)						
Female	96 (31.9)	67 (69.8)	29 (30.2)	0.851	1	0.356
Male	205 (68.1)	132 (64.4)	73 (35.6)			
Income status, *n* (%)				0.507	2	0.776
Low	35 (11.6)	24 (68.6)	11 (31.4)			
Middle	159 (52.8)	107 (67.3)	52 (32.7)			
High	107 (35.5)	68 (63.6)	39 (36.4)			
Marital status, *n* (%)				0.703	2	0.704
Married	82 (27.2)	132 (64.7)	72 (35.3)			
Single	204 (67.8)	56 (68.3)	26 (31.7)			
Other	15 (5.0)	11 (73.3)	4 (26.7)			
Employment status, *n* (%)				0.623	3	0.891
Employed	212 (70.4)	142 (67.0)	70 (33.0)			
Unemployed	9 (3.0)	5 (55.6)	4 (44.4)			
Student	75 (24.9)	49 (65.3)	26 (34.7)			
Retired	5 (1.7)	3 (60.0)	2 (40.0)			
Living arrangement, *n* (%)				2.281	3	0.516
Living Alone	79 (26.2)	48 (60.8)	31 (39.2)			
With family	83 (27.6)	58 (69.9)	25 (30.1)			
With parents	109 (36.2)	71 (65.1)	38 (34.9)			
Other	30 (10.0)	22 (73.3)	8 (26.7)			
Type of sport, *n* (%)				7.570	7	0.372
Fitness	95 (31.6)	58 (61.1)	37 (38.9)			
Wrestling	39 (13.0)	23 (59.0)	16 (41.0)			
Football (socker)	44 (14.6)	33 (75.0)	11 (25.0)			
Volleyball	37 (12.3)	27 (73.0)	10 (27.0)			
Athletics	24 (8.0)	16 (66.7)	8 (33.8)			
Swimming	6 (2.0)	3 (50.0)	3 (50.0)			
Weightlifting	5 (1.7)	5 (100)	0			
Other	51 (16.9)	34 (66.7)	17 (33.3)			
Duration of sports participation, years (mean ± SD)	8.83 (7.18)	9.07 (7.43)	8.37 (6.67)	0.803	299	0.422
Sport type, *n* (%)				2.665	1	0.103
Individual	209 (69.4)	132 (63.2)	77 (36.8)			
Team	92 (30.6)	67 (72.8)	25 (27.2)			
Sport level				0.087	1	0.768
Amateur	187 (62.5)	125 (66.8)	62 (33.2)			
Professional	112 (37.5)	73 (65.2)	39 (34.8)			
Gym membership, *n* (%)				3.381	2	0.184
Monthly	72 (23.9)	46 (63.9)	26 (36.1)			
Annual	141 (46.8)	88 (62.4)	53 (37.6)			
Other	88 (29.2)	65 (73.9)	23 (26.1)			
Smoking status				7.060	2	**0.029**
Yes	73 (24.3)	47 (64.4)	26 (35.6)			
No	205 (68.1)	131 (63.9)	74 (36.1)			
Former smoker	23 (7.6)	21 (91.3)	2 (8.7)			

SD, standard deviation.

The mean age was 29.0 ± 7.9 years for the overall group, 27.9 ± 6.8 years for participants with alexithymia, and 29.6 ± 8.3 years for those without alexithymia. The two groups were comparable in age, education, gender, income, marital status, employment, and living arrangements (all *p* > 0.05). Smoking was the only variable that differed: smokers were more frequent among participants without alexithymia (64.4%) than among those with alexithymia (35.6%). (*χ^2^* = 7.060, *p* = 0.029).

Regarding exercise profiles, no significant differences were found between the groups for the type of sport practiced, duration of sports participation, engagement in individual versus team sports, amateur versus professional status, or gym membership characteristics (all p > 0.05).

### 3.2 Clinical measures according to alexithymia status

Table 2 shows the clinical comparisons between participants with and without alexithymia. Those with alexithymia scored higher on every EDS-21 subscale, and all differences were statistically significant. Specifically, they reported higher scores for time and exercise preference (*p* < 0.001), lack of control (*p* < 0.001), withdrawal effects (*p* < 0.001), tolerance (*p* < 0.001), and continuance (*p* < 0.001). The EDS-21 total score was also significantly higher in the alexithymia group than in the non-alexithymia group (*p* < 0.001). There was no significant difference in ACE-Q total scores between groups (*p* = 0.442).

**Table 2:** Comparison of clinical measurement of the participants according to alexithymia.

Variable	Total participant (*n* = 301)	Alexithymia		*t*/U value	df value	*p* value
		No (*n* = 199)	Yes (*n* = 102)			
EDS-21, (mean ± SD)						
Time and exercise preference	23.44 ± 9.91	21.44 ± 8.40	27.36 ± 11.40)	−5.104	299	<0.001
Lack of control	7.65 ± 3.81	6.99 ± 3.53	8.96 ± 4.31)	−4.365	299	<0.001
Withdrawal effects	9.23 ± 3.70	8.53 ± 3.50	10.58 ± 3.73)	−4.697	299	<0.001
Tolerance	9.51 ± 3.91	8.82 ± 3.62	10.85 ± 4.12)	−4.382	299	<0.001
Continuance	8.51 ± 3.91	7.75 ± 3.45	9.99 ± 4.33)	−4.856	299	<0.001
Total score	58.25 ± 22.21	53.38 ± 18.87	67.75 ± 25.08)	−5.573	299	<0.001
ACE total score, median (IQR)	1 (2.00)	1 (2.00)	1 (2.00)	9,932	—	0.442
TAS-20, (mean ± SD)						
DIF	15.34 ± 5.36	12.39 ± 3.45	21.09 ± 4.50	−18.60	299	<0.001
DDF	13.90 ± 2.98	12.59 ± 2.41	16.45 ± 2.25	−13.39	299	<0.001
EOT	26.31 ± 4.13	25.21 ± 3.85	28.46 ± 3.82	−6.942	299	<0.001
Total score	55.55 ± 9.57	50.20 ± 5.72	66.01 ± 6.38	−21.78	299	<0.001

ACE, adverse childhood experience; DDF, difficulty describing feelings; DIF, difficulty identifying feelings; EDS-21, exercise dependence scale-21; EOT, externally oriented thinking; TAS-20, Toronto alexithymia scale-20.

As expected based on the group definition, participants with alexithymia had significantly higher scores on all TAS-20 subscales, including DIF (*p* < 0.001), DDF (*p* < 0.001), and EOT (*p* < 0.001), as well as higher TAS-20 total scores (p < 0.001).

### 3.3 Correlation between exercise dependence, alexithymia dimensions, and childhood trauma

Table 3 reports correlations among exercise dependence (EDS-21) dimensions, alexithymia subscales (TAS-20), and CT (ACE-Q). All EDS-21 subscales showed positive associations with DIF, including time and exercise preference (*r* = 0.343, *p* < 0.001), lack of control (*r* = 0.284, *p* < 0.001), withdrawal effects (*r* = 0.296, *p* < 0.001), tolerance (*r* = 0.230, *p* < 0.001), and continuance (*r* = 0.318, *p* < 0.001). The EDS-21 total score was similarly moderately correlated with DIF (*r* = 0.345, *p* < 0.001).

**Table 3:** Association between exercise dependence, alexithymia dimensions, and childhood trauma.

Variable	Statistic	DIF	DDF	EOT	TAS-20 Total
Time and exercise preference	*r*	0.343	0.234	0.052	0.298
	*p*	<0.001	<0.001	0.369	<0.001
Lack of control	*r*	0.284	0.187	0.074	0.258
	*p*	<0.001	0.001	0.202	<0.001
Withdrawal effects	*r*	0.296	0.190	0.127	0.289
	*p*	<0.001	<0.001	0.028	<0.001
Tolerance	*r*	0.230	0.110	0.104	0.215
	*p*	<0.001	0.056	0.072	<0.001
Continuance	*r*	0.318	0.169	0.090	0.279
	*p*	<0.001	0.003	0.118	<0.001
EDS-21 Total score	*r*	0.345	0.224	0.095	0.314
	*p*	<0.001	<0.001	0.101	<0.001
ACE Total score	*r*	0.263	0.081	0.108	0.227
	*p*	<0.001	0.159	0.061	<0.001

ACE, adverse childhood experience; DDF, difficulty describing feelings; DIF, difficulty identifying feelings; EDS-21, exercise dependence scale-21; EOT, externally oriented thinking; TAS-20, Toronto alexithymia scale-20.

Several EDS-21 subscales were also positively correlated with DDF, including time and exercise preference (*r* = 0.234, *p* < 0.001), lack of control (*r* = 0.187, *p* = 0.001), withdrawal effects (*r* = 0.190, *p* < 0.001), and continuance (*r* = 0.169, *p* = 0.003). The EDS-21 total score likewise showed a significant association with DDF (*r* = 0.224, *p* < 0.001). By contrast, associations between EDS-21 subscales and EOT were generally small and non-significant, except for withdrawal effects, which showed a modest but statistically significant correlation. (*r* = 0.127, *p* = 0.028).

All EDS-21 subscales and the EDS total score were significantly positively correlated with TAS-20 total scores (all *p* < 0.001).

CT, as measured by the ACE-Q total score, was significantly correlated with DIF (*r* = 0.263, *p* < 0.001) and TAS-20 total score (*r* = 0.227, *p* < 0.001), whereas correlations with DDF (*r* = 0.081, *p* = 0.159) and EOT (*r* = 0.108, *p* = 0.061) were not statistically significant.

### 3.4 The role of alexithymia in the association between childhood trauma and exercise dependence

**Figure 1:**
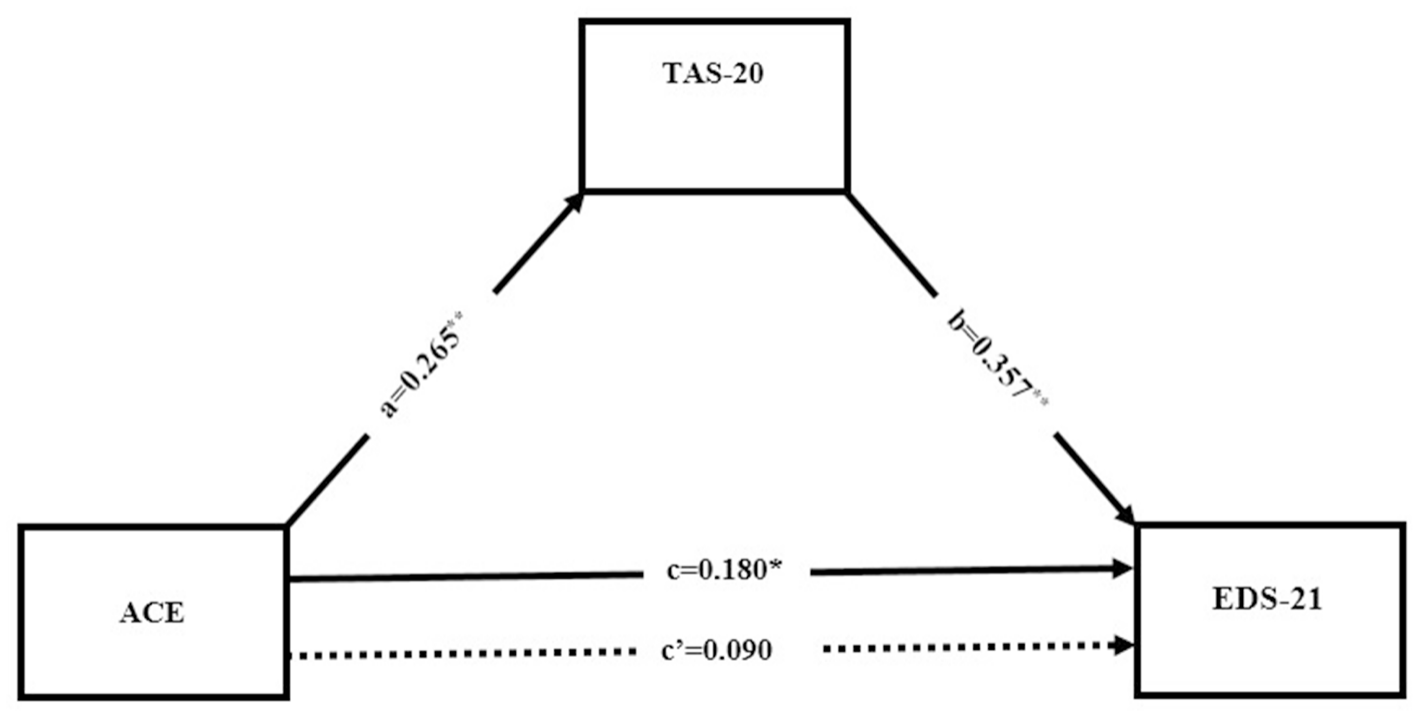
Mediation model examining the indirect effect of childhood trauma on exercise dependence via alexithymia. ACE, adverse childhood experiences (ACE-Q total score); TAS-20, Toronto Alexithymia Scale-20 total score; EDS-21, Exercise Dependence Scale-21 total score. Standardized coefficients are shown. *p < 0.05; **p < 0.001.

A regression-based mediation model (ACE-Q → TAS-20 → EDS-21) was tested to examine whether alexithymia was involved in the association between ACE and EA (Figure 1).

In the total effect model, the ACE-Q total score was significantly associated with EDS-21 total score (path c: *β* = 0.180, *p* = 0.002; *B* = 1.001, SE = 0.322, *t* = 3.110). ACE-Q total score was also significantly associated with TAS-20 total score (a: *β* = 0.265, *p* < 0.001; B = 0.833, SE = 0.193, *t* = 4.318). In addition, TAS-20 total score was significantly associated with EDS-21 total score (b: *β* = 0.357, *p* < 0.001; B = 0.618, SE = 0.136, *t* = 4.551).

TAS-20 total score was added to the model, and the direct ACE-Q-EDS-21 association no longer reached statistical significance (c′: *β* = 0.090, *p* = 0.133; B = 0.489, SE = 0.325, *t* = 1.502). This finding indicates that the ACE-Q–EDS-21 relationship is statistically insignificant when alexithymia is taken into account.

The indirect effect of ACE-Q on EDS-21 through TAS-20 was significant (*β* = 0.095, B = 0.515, 95% CI 0.194–0.836), based on bias-corrected bootstrapping with 5,000 resamples.

## 4 Discussion

This study examined the relationships among CT, alexithymia, and EA severity in regularly exercising adults, with alexithymia placed as an intermediary variable. Participants with alexithymia reported greater exercise dependence across all EDS-21 domains than those without alexithymia. Although no significant group differences were observed in CT exposure as measured by ACE-Q scores, correlation analysis revealed that CT was significantly associated with the DIF dimension of alexithymia. Mediation analysis results suggested an indirect association between CT and EA via alexithymia. CT showed a significant total effect on EA, but the direct association was no longer significant after alexithymia was entered into the model.

The finding that individuals with alexithymia exhibit higher EA severity supports the theoretical view that considers alexithymia as a risk factor for addictive behaviors (Orsolini, 2020; Blinka et al., 2024; Mahapatra and Sharma, 2018). This is consistent with broader evidence indicating that emotional dysregulation can lead to addictive behaviors, which may serve as a maladaptive way to cope with distressing emotions (González-Roz et al., 2024; Morie et al., 2016). Our results directly support recent studies that highlight a significant positive association between alexithymia and EA (Gori et al., 2021; Bossard and Miller, 2009; Lyvers et al., 2021). Exercise can improve mood and produce physiological changes that help people recover from stress (Bernstein and McNally, 2018). For some individuals with alexithymia, exercise may serve as a way of reducing poorly differentiated, hard-to-name distress. When it becomes the primary means of managing this discomfort, the behavior can be reinforced through repetition and may gradually develop into a more compulsive pattern, with increasing risk of dependence (Allegre et al., 2007; Szabo and Demetrovics, 2022). Our correlational findings further clarify this connection. The emotional aspect of alexithymia, specifically the challenges in identifying and describing emotions, was most closely linked to EA symptoms. In contrast, the cognitive trait of externally oriented thinking did not show connections, with the exception of withdrawal effects. Taken together, the results point to emotional-processing difficulties, not general cognitive traits, as the key drivers of addictive exercise. Supporting evidence also indicates that emotion regulation helps explain this relationship (Sweetnam and Flack, 2023).

The present findings are not entirely consistent with prior literature, and this warrants discussion. Colledge et al. (2022) found that individuals at risk for EA reported higher CT scores than those not at risk (Colledge et al., 2022), our study did not find a significant difference in cumulative CT exposure between participants with and without alexithymia. The apparent discrepancy becomes more intelligible in light of our main mediation result. Even though we did not observe a clear between-group difference, the significant positive association between overall CT burden (ACE-Q scores) and the DIF subscale is consistent with the well-established link between early adversity and reduced access to internal emotional cues (Kick et al., 2024; Ditzer et al., 2023; Cerqueira and Almeida, 2023). This specificity is supported by network studies showing that emotional abuse and DIF form closely connected, central nodes within the trauma–alexithymia structure (Yang et al., 2024). Evidence also indicates that DIF emerges as the most robust unique predictor of later emotional distress (Preece et al., 2024). Because trauma levels are relatively lower in this community sample, the association-based signal may be clearer than any between-group mean difference when it comes to CT’s finer effects on emotional development. The absence of a significant correlation with DDF may reflect a difference in what trauma impacts most. Identifying emotions from within and articulating them verbally appear to be distinct processes. Verbal description may be more strongly shaped by subsequent interpersonal and communicative experiences.

The primary contribution of this study is the empirical evidence showing that alexithymia acts as a mediator in the link between CT and EA. This finding highlights alexithymia not just as a correlate but also as an associated factor and a crucial psychological mechanism that may explain the association between early adversity and maladaptive behaviors. This pathway is already well supported in the literature. Multiple studies have shown that alexithymia mediates the link between adverse childhood experiences and broader psychopathology (Kick et al., 2024; Cooper et al., 2024; Karaca Dinç et al., 2021). The present findings suggest that the same mechanism may also be relevant for behavioral addiction. A recent study showed that the connection between CT and EA is influenced by broader issues in emotion regulation and unmet basic psychological needs (Arayici and Tekinsav Sutcu, 2025) Our results point to alexithymia as a simple but important piece of the puzzle: difficulty noticing and naming what one feels. One possibility is that CT may disrupt the normal learning of emotional awareness and self-regulation, and that this disruption shows up later as alexithymic features. This condition may then increase the likelihood of engaging in excessive exercise as a compensatory, yet maladaptive, regulatory strategy, ultimately contributing to addictive behaviors. This model is supported by our finding that the affective components of alexithymia (difficulty identifying and describing feelings), which were most strongly linked to CT and EA, are central to this dysregulation. This model is consistent with previous studies linking CT and alexithymia to other behavioral addictions, including problematic internet use and substance use (Sutherland et al., 2022; Wang et al., 2025; Garami et al., 2019). Taken together, these findings suggest that emotion regulation difficulties stemming from early traumatic experiences may be influential across diagnostic categories.

When interpreting these findings, it is important to acknowledge several limitations. The cross-sectional nature of the study prevents definitive causal conclusions. Although our model is grounded in theoretical foundations, longitudinal research is necessary to verify the sequence in which CT leads to alexithymia, which, in turn, increases susceptibility to EA. Data relied on self-report measures, which can be susceptible to recall bias, especially when reporting retrospective adverse childhood experiences. Additionally, responses on scales for alexithymia and EA may be influenced by social desirability or an individual’s current mood states. The study sample consisted of individuals actively engaged in exercise from sports centers in one Turkish city, which may limit the generalizability of the findings to clinical populations seeking treatment for EA, non-exercisers, or other cultural contexts. The sample was predominantly male (68.1%), which may limit the generalizability of the findings to female exercisers. Although no significant gender difference in alexithymia status was observed in the present sample, gender differences in alexithymia, exercise addiction, and childhood trauma exposure have been documented in the broader literature. Men have been reported to score higher on alexithymia and to show higher rates of exercise addiction, while gender differences have also been described in the type and impact of childhood trauma (Mendia et al., 2024; Alcaraz-Ibáñez et al., 2022; Prachason et al., 2023). The present study did not examine gender as a moderator of the mediation model. Future research should include more gender-balanced samples and explicitly test whether gender moderates the associations among childhood trauma, alexithymia, and exercise addiction severity. While the study identified alexithymia as a significant mediator, other important variables were not assessed. This model has not taken into account potential confounding factors such as co-occurring symptoms of depression or anxiety that may be linked to CT and addictive behaviors (Colledge et al., 2022; Huh et al., 2017; Fuchshuber et al., 2018). Additionally, other psychological factors such as attachment styles, family dynamics, or impulsivity may also play a mediating or moderating role in the relationship between CT and EA (Arayici and Tekinsav Sutcu, 2025; Migalova et al., 2025). These variables were not included in the present study in order to maintain focus on the primary mediation pathway. Examining their independent and combined contributions to the CT–alexithymia–EA model represents an important direction for future research. Future research should employ longitudinal designs to establish temporal precedence, incorporate clinician-administered interviews, include more diverse and clinical samples, and explore whether interventions directly targeting alexithymia result in decreased severity of EA.

Despite these limitations, the findings carry meaningful clinical and theoretical implications. Theoretically, the present results support the view that alexithymia functions not merely as a personality trait but as a psychologically active mechanism linking early adversity to later maladaptive behavior. This positions alexithymia as a transdiagnostic factor worthy of attention across behavioral addiction research, extending beyond exercise to other domains such as problematic internet use and compulsive behaviors. From a research perspective, these findings suggest that future studies examining EA should routinely assess alexithymia and childhood trauma history, and consider testing more complex models incorporating multiple mediators such as emotion regulation strategies, attachment, and interoceptive awareness.

The link between CT and EA through alexithymia highlights the importance of looking beyond behavioral assessments. In clinical practice, individuals presenting with EA should be screened for alexithymia and past childhood adversity. This reframes EA not merely as compulsive behavior but as a potential maladaptive coping strategy for managing undifferentiated emotional distress rooted in early trauma. Treatment approaches may therefore benefit from integrating interventions that target emotion-processing deficits. Systematic reviews have shown that mindfulness-based interventions can effectively reduce alexithymia (Norman et al., 2019). Initial evidence also suggests that Dialectical Behavior Therapy (DBT) may improve the ability to identify emotional states (Salles et al., 2023). Including approaches that emphasize developing emotional awareness, recognizing emotions, and understanding their roles could be significant in addressing the alexithymia aspects of EA.

## 5 Conclusion

This study suggests that alexithymia may present a relevant psychological factor in the association between CT and EA. Consequently, assessment and treatment strategies for EA may benefit from considering emotional awareness and emotional processing difficulties, particularly in individuals with a history of childhood trauma. Longitudinal studies are needed to clarify the temporal relationships among these variables.

## Data Availability

The original contributions presented in the study are included in the article/supplementary material, further inquiries can be directed to the corresponding author.
